# Dataset from the global phosphoproteomic mapping of early mitotic exit in human cells

**DOI:** 10.1016/j.dib.2015.08.010

**Published:** 2015-08-24

**Authors:** Samuel Rogers, Rachael A. McCloy, Benjamin L. Parker, Rima Chaudhuri, Velimir Gayevskiy, Nolan J. Hoffman, D. Neil Watkins, Roger J. Daly, David E. James, Andrew Burgess

**Affiliations:** a The Kinghorn Cancer Center, Garvan Institute of Medical Research, Darlinghurst, NSW 2010, Australia; b The Charles Perkins Centre, School of Molecular Bioscience and Sydney Medical School, The University of Sydney, NSW 2006, Australia; c St. Vincent׳s Clinical School, Faculty of Medicine, UNSW, Darlinghurst, NSW, Australia; d Department of Thoracic Medicine, St Vincent׳s Hospital, Darlinghurst, NSW 2010, Australia; e Department of Biochemistry and Molecular Biology, School of Biomedical Sciences Monash University, Clatyon, VIC 3800, Australia

## Abstract

The presence or absence of a phosphorylation on a substrate at any particular point in time is a functional readout of the balance in activity between the regulatory kinase and the counteracting phosphatase. Understanding how stable or short-lived a phosphorylation site is required for fully appreciating the biological consequences of the phosphorylation. Our current understanding of kinases and their substrates is well established; however, the role phosphatases play is less understood. Therefore, we utilized a phosphatase dependent model of mitotic exit to identify potential substrates that are preferentially dephosphorylated. Using this method, we identified >16,000 phosphosites on >3300 unique proteins, and quantified the temporal phosphorylation changes that occur during early mitotic exit (McCloy et al., 2015 [1]). Furthermore, we annotated the majority of these phosphorylation sites with a high confidence upstream kinase using published, motif and prediction based methods. The results from this study have been deposited into the ProteomeXchange repository with identifier PXD001559. Here we provide additional analysis of this dataset; for each of the major mitotic kinases we identified motifs that correlated strongly with phosphorylation status. These motifs could be used to predict the stability of phosphorylated residues in proteins of interest, and help infer potential functional roles for uncharacterized phosphorylations. In addition, we provide validation at the single cell level that serine residues phosphorylated by Cdk are stable during phosphatase dependent mitotic exit. In summary, this unique dataset contains information on the temporal mitotic stability of thousands of phosphorylation sites regulated by dozens of kinases, and information on the potential preference that phosphatases have at both the protein and individual phosphosite level. The compellation of this data provides an invaluable resource for the wider research community.

Specifications Table

**Table:** 

Subject area	Cell biology
More specific subject area	Phosphoproteomics and Mitosis
Type of data	MS data and annotations, western blot, time-lapse microscopy, immunofluorescence
How data was acquired	Mass spectrometry (LTQ-Orbitrap Velos Pro, Thermo Fisher Scientific), Leica TCS SP8 MP confocal microscope
Data format	Raw (.raw,index,.apl), filtered, and analyzed data (.txt and.xlsx)
Experimental factors	SILAC labeled Nocodazole arrested HeLa cells, treated with the protease inhibitor MG132, followed with (heavy) or without (light) the Cdk1 inhibitor RO3306.
Experimental features	Mitotic arrested and mitotic exit samples were lysed, mixed 1:1, peptides were digested with trypsin and fractionated using strong cation exchange. Phosphopeptides were enriched using TiO2, and samples were analyzed by LC-MS/MS.
Data source location	Sydney, Australia
Data accessibility	All raw MaxQuant output data is available in the PRIDE repository http://www.ebi.ac.uk/pride/archive/projects/PXD001559. Annotated spectra can be viewed using the free MS-viewer http://prospector2.ucsf.edu with the search key gsmtp1s5q7

Value of the data

**Table:** 

• Temporal, quantitative data on over 16,000 phosphorylation sites on more than 3300 proteins. • Majority of phosphorylation sites have been annotated with known and/or predicted upstream kinase/s, in an easy to use excel spreadsheet, providing an excellent resource for the wider research community. • Identification of several new motifs for the major mitotic kinases that correlate with phosphosite stability. • These motifs could be used to predict the potential phosphorylation stability of specific phosphorylated residues of interest.

## 1 Data

**Fig.1:**
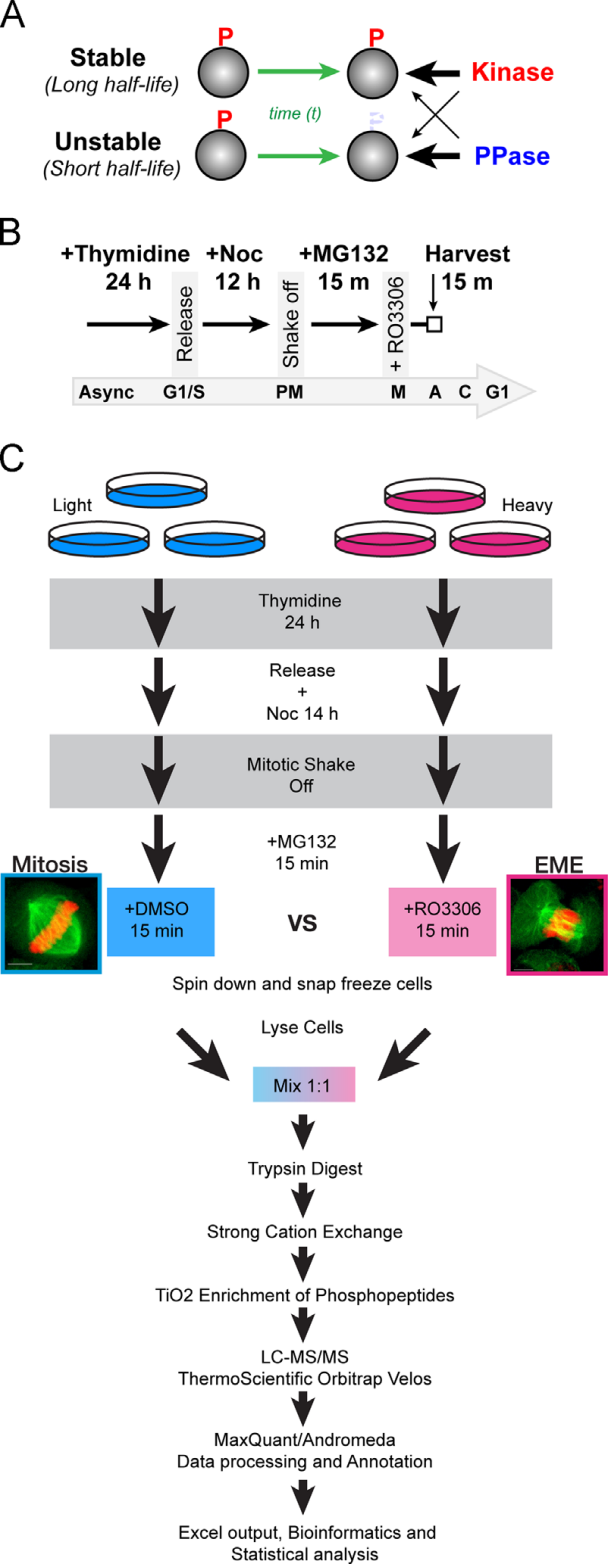
(a) Shown is a simplistic model for creating stable and unstable phosphorylation sites by altering the preference that each kinase and phosphatase pair has for a specific phosphosite. Thick arrows (black) indicate a stronger preference compared to thin arrows. For example, sites that are preferentially dephosphorylated by a phosphatase will be unstable. (B) Schematic diagram of method for producing highly synchronized HeLa cells undergoing phosphatase dependent mitotic exit. (C) Schematic diagram detailing SILAC metabolic labeling of mitotic and early (phosphatase dependent) mitotic exit samples. This was then followed by peptide digestion, fractionation, phosphopeptide enrichment, quantification by LC-MS/MS, peptide identification and annotation using MaxQuant environment and finally statistical and bioinformatics analysis.

Phosphorylation is a dynamic modification, and therefore to fully understand the meaning of a specific phosphorylation, its half-life must be known. The stability is an output of the activity of the regulatory kinase and phosphatase (Fig. 1A). In order to understand the dynamic nature of phosphorylation sites, we took advantage of the fact that during mitosis over 75% of the human proteome (>7000 proteins) is phosphorylated, with those proteins phosphorylated on the majority of all potential phosphorylation sites [2]. As cells exit mitosis these phosphorylations are removed in a highly organized, sequential manner [3]. Therefore, mitotic exit provides an excellent experimental system to rapidly analyze the temporal dynamics of phosphorylation. We recently performed a global phosphoproteomics analysis comparing mitosis to early mitotic exit [1], and here we present detailed methods and additional data from this study. This additional information can be used by the wider research community to infer a potential function of a phosphorylation sites based on our reported mitotic temporal dynamics, or as predictive tool for the stability of a novel phosphorylation based amino acids surrounding the phosphosite.

## 2 Experimental design, materials, and methods

### 2.1 Cell synchrony

In order to analyze temporal events during mitotic exit, highly synchronized cell cultures are needed. To achieve this, we utilized a two-step synchronization protocol using HeLa cells (Fig. 1B). Briefly, cells were seeded at approximately 70% confluence on large 15 cm plates. They were allowed to attached and were then treated with 1 mM Thymidine for 24 h. Cells were released from G1/S arrest by washing 3 times with pre-warmed media, and then re-adding fresh media supplemented with 25 µM 2′-Deoxycytidine (Santa Cruz #sc-231247). To capture cells in prometaphase (PM), G1/S released cells were treated with 100 ng/ml of Nocodazole for 14 h. Further enrichment of mitotic cells was achieved by gentle shake-off, with floating cells pooled into 50 ml falcon tubes. Mitotic cells were then treated with 25 µM MG132 for 15 min to prevent protein degradation and to ensure cells did not progress past metaphase. To trigger synchronized phosphatase dependent mitotic exit, enriched mitotic cells were treated with the Cdk1 inhibitor RO3306 (10 µM).

## 3 SILAC labeling

HeLa cells were SILAC-labeled by culturing in DMEM where the natural “light” Lysine and Arginine were replaced by “heavy” isotope-labeled amino acids ^13^C_6_^15^N_4_-l-Arginine (Arg 10) and ^13^C_6_^15^N_2_-l-Lysine (Lys 8) (Silantes GmBH), which was supplemented with 10% dialyzed FBS and 4 mM glutamine. To ensure complete labeling of >97%, cells were cultured for approximately six doublings in heavy or light media, with fresh media replaced every two days and sub-culturing performed when cells reached 90% confluence. After labeling, cells were synchronized as per Fig. 1B. Mitotic cells were enriched by shake off, and both light and heavy labeled samples were treated with 25 µM MG132 for 15 min. Heavy labeled samples were then treated with 10 µM RO3306 (RO) for a further 15 min, with both samples then harvested by centrifugation at 4 °C (Fig. 1C). Three biological replicates were prepared, and in one replicate, the heavy/light labels were switched to provide an internal labeling control.

## 4 Mass spectrometry

Cells were lysed in urea lysis buffer (2.5 mM sodium pyrophosphate, 1 mM β-glycerol phosphate, 1 mM sodium orthovanadate, 1 mM tris (2-carboxy- ethyl) phosphine (TCEP), 1 mM EDTA, 8 M urea and 20 mM HEPES), sonicated, and then iodoacetamide was added to 100 mM. Protein concentration was determined by the Bradford assay (Thermo Fisher Scientific, Scoresby, VIC, Australia). Samples were then mixed 1:1 (light:heavy) based on quantification of total protein and digested in-solution with modified TPCK treated trypsin (Promega). Peptides were desalted on C18 solid-phase extraction columns and separated into 9 fractions by strong-cation exchange (SCX) using the ÄKTApurifier (GE Healthcare) followed by TiO_2_ phosphopeptide enrichment as previously described [4,5]. Peptides were resuspended in 0.5% acetic acid and loaded onto a laser-drawn ~30 cm, 75 µm I.D. fused silica column, packed in house with 3 µm ReproSil Pur-120 C18AQ beads (Dr. Maisch, Germany) using an Easy nLC-II (ThermoFisher Scientific) and eluted with a linear gradient of 0–30% acetonitrile containing 0.5% acetic acid. Phosphopeptides were analyzed on an LTQ-Orbitrap Velos Pro (Thermo Fisher Scientific) (Fig. 1C). A precursor MS scan (350–1650 m/z) was acquired in the Orbitrap at a resolution of 60,000 followed by data-dependent CID MS/MS in the LTQ of up to 20 most abundant precursor ions.

## 5 Peptide identification using MaxQuant

Mass spectra were processed with version 1.2.7.4 of the MaxQuant software package (http://www.maxquant.org) using default settings with the inclusion of match between runs option. Peptides were assigned incorporating modified arginine-10 and lysine-6, with a maximum of 2 missed cleavages, using the fixed modification carboxyamidomethylation, and variable methionine oxidation and STY phosphorylation. Database searching was performed using the Andromeda search engine integrated into the MaxQuant environment [6] against the complete human proteome containing 88,820 sequence entries (UniProt release-2013_06, ftp://ftp.uniprot.org). Precursor mass tolerance was set at 20 ppm for initial search, fragmentation peptide to 0.6 Da. To ensure high quality protein identifications, MS spectra were also searched against a reverse database of a similar size with the false discovery rate limited to <1%. Known contaminants identified by MaxQuant were filtered out of the initial dataset.

## 6 Description of dataset contained on public repositories

We have uploaded all the raw mass spectrometry data files and MaxQuant output files necessary to reanalyze the complete dataset to the ProteomeXchange Consortium (http://proteomecentral.proteomexchange.org) via the PRIDE partner repository [7] with the identifier PXD001559. Annotated spectra can be viewed using the free MS-viewer [8] (http://prospector2.ucsf.edu) with the search key gsmtp1s5q7. In addition, a summary of this data, in an easy to use excel spreadsheet, is provided with this manuscript (Supplementary Table S1).

## 7 Statistical analysis

To ensure that only highly confident protein identifications were reported, phosphosite identifications were filtered in excel for those with a localization probability greater than 0.75, a minimum MaxQuant score of 30 and a maximum posterior error probability of 1%. A fold change cut off of ≥4 (log_2_ ratio ≥+2 or ≤−2) was used to identify increased and dephosphorylated phosphopeptides, respectively. A moderated *t*-test was used to identify phosphosites that are significantly up or down-regulated using Linear Models for Microarray and RNA-Seq Data (LIMMA) package in R [9]. LIMMA allows for global variance shrinkage using an empirical Bayes model. Identified sites were then corrected for multiple hypothesis testing using the Benjamini and Hochberg method (controlling for 5% false discovery rate). Phosphopeptides were considered to be stable if they were non-significant proteins (adj.*p*.value>0.05), and had log_2_ ratios between −0.25 and +0.25 with a standard deviation <0.5. Supplemental Table S1 contains a summary of all the phosphosites identified, along with moderated *t*-statistics, *p*-values and adjusted *p*-values for all phosphosites.

## 8 Data analysis

**Fig. 2:**
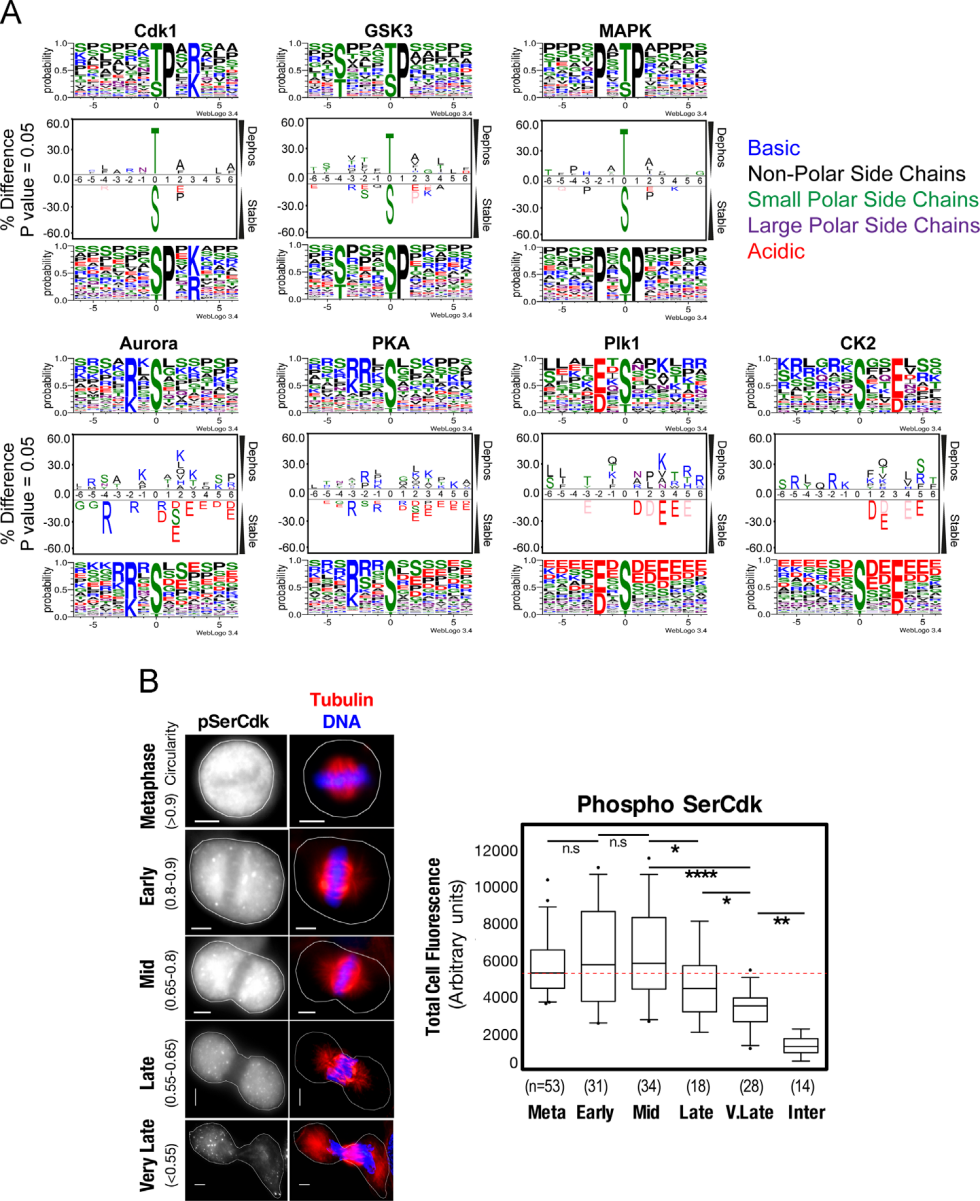
(A) Dephosphorylated (log_2_ <−2) and stable stable (log_2_ −0.25 to +0.25) S/T–P phosphopeptides for each of the major mitotic kinases were compared using IceLogo and WebLogo motif analysis software. Differentially enriched amino acids can be identified by the increasing letter size, and distance away from center. (B) Quantitative immunofluorescence of pSerCdk levels in individual cells undergoing phosphatase dependent mitotic exit. The levels of pSerCdk are relatively stable during exit with no significant loss in staining is observed until cells have progressed to the late phases of exit, confirming the motif observed in (A). Scale bars=5 µM. Shown are box plots with 5–95% confidence intervals. Significant *p*-values from 1-way ANOVA with Newman–Keuls correction for multiple comparisons are shown (⁎=<0.01, ⁎⁎=<0.001, ⁎⁎⁎⁎=<0.00001, n.s=not significant).

Annotation of upstream kinase was done using the reported minimal consensus motifs for each kinase [1] and using KinomeXplorer (http://kinomexplorer.info) [10]. This information is annotated in Supplementary Table S1. Simple data analysis of this table can be performed using Microsoft Excel and the filter function. Sequences for each kinase for the statistically significant dephosphorylated, and stable phosphosites were analyzed using motif enrichment analysis with Icelogo [11] and WebLogo 3 [12]. The results of this analysis are shown in Fig. 2A. Briefly, acidic residues (D, E) upstream (right) of the phosphorylation site are more commonly associated with stable phosphosites (Fig. 2A). Based on our simplistic model (Fig. 1A), these acidic residues could inhibit or reduce the preference of phosphatases for these phosphorylation sites, thereby creating a stable (long half-life) phosphorylation (Fig. 1A).

## 9 Quantitative immunofluorescence staining

To validate the motifs observed in Fig. 2A, we performed quantitative immunofluorescence staining of cells undergoing phosphatase dependent mitotic exit (Fig. 2B). Cells, grown on Histogrip (Invitrogen) coated glass coverslips, were synchronized as per Fig. 1B, and harvested using ice cold 100% methanol at 0 min (Metaphase), 15 min (Early), 30 min (Mid) 45 min (Late) and 60 min (Very Late) post addition of the Cdk1 inhibitor RO33306. Fixed cells were washed and blocked (3% BSA, 0.1% Tween 20 in PBS) for 30 min, then incubated with primary antibodies for pSerCdk (#2324, Cell Signaling Technologies) and β-tubulin [13] for 2 h at room temperature in blocking solution. Mouse and Rabbit secondary Alexa 488 and 594 (Invitrogen) antibodies along with DAPI were used to visualize pSerCdk, microtubules, and DNA respectively. Images were captured using a Leica DM5500 microscope coupled with a Coolsnap HQ2 camera, using a Leica 100X or 40X APO 1.4 lens, powered by Leica LAS AF v3 software. To quantify pSerCdk levels in cells, a single in-focus plane was acquired using identical microscope settings for all conditions. Analysis was performed using Image J (v1.48, NIH) an outline drawn around each cell and circularity, area, mean fluorescence measured, along with several adjacent background readings. The Total Corrected Cellular Fluorescence (TCCF)=Integrated Density – (Area of selected cell×Mean fluorescence of background readings), was calculated. Box-plots and statistical analysis (ordinary one-way ANOVA, with Newman–Keuls correction for multiple comparisons) were performed using GraphPad Prism 6. For all β-tubulin and DAPI, 0.3 µm *z*-sections were taken, de-convolved, and displayed as 2D maximum projections using Image J. False coloring and overlays were performed using Adobe Photoshop CC 2015 software.

## Footnotes

Appendix A Supplementary data associated with this article can be found in the online version at doi:10.1016/j.dib.2015.08.010.
