# Supplementary material for: Dataset from the global phosphoproteomic mapping of early mitotic exit in human cells
Source: Data Brief. 2015 Aug 24;5:45–52. doi: 10.1016/j.dib.2015.08.010 (PMC4564385; doi:10.1016/j.dib.2015.08.010)
Supplement: Supplementary file 1 — Supplementary Data [file mmc1.pdf]

AUTHOR DECLARATION

I wish to confirm that there are no known conflicts of interest associated with this publication and there has been no significant financial support for this work that could have influenced its outcome.

I confirm that the manuscript has been read and approved by all named authors and that there are no other persons who satisfied the criteria for authorship but are not listed. I further confirm that the order of authors listed in the manuscript has been approved by all of authors.

I confirm that authors have given due consideration to the protection of intellectual property associated with this work and that there are no impediments to publication, including the timing of publication, with respect to intellectual property. In so doing I confirm that I have followed the regulations of our institutions concerning intellectual property.

All authors understand that the Corresponding Author is the sole contact for the Editorial process (including Editorial Manager and direct communications with the office). He is responsible for communicating with the other authors about progress, submissions of revisions and final approval of proofs. All authors confirm that they have provided a current, correct email address which is accessible by the Corresponding Author and which has been configured to accept email from a.burgess@garvan.org.au.

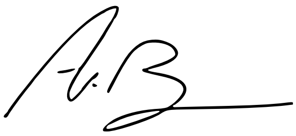

Andrew Burgess

August 4, 2015
